# Relationship between Body Mass Index and Percent Body Fat in Vietnamese: Implications for the Diagnosis of Obesity

**DOI:** 10.1371/journal.pone.0127198

**Published:** 2015-05-27

**Authors:** Lan T. Ho-Pham, Thai Q. Lai, Mai T. T. Nguyen, Tuan V. Nguyen

**Affiliations:** 1 Bone and Muscle Research Division, Faculty of Applied Sciences, Ton DucThang University, Ho Chi Minh City, Vietnam; 2 Department of Internal Medicine, Pham Ngoc Thach University of Medicine, Ho Chi Minh City, Vietnam; 3 Department of Rheumatology, People’s Hospital 115, Ho Chi Minh City, Vietnam; 4 Department of Medical Ethic—Behavioral Science, Pham Ngoc Thach University of Medicine, Ho Chi Minh City, Vietnam; 5 Osteoporosis and Bone Biology, Garvan Institute of Medical Research, Sydney, Australia; 6 School of Public Health and Community Medicine, UNSW Australia, Sydney, Australia; 7 Centre for Health Technologies, University of Technology, Sydney, Australia; National Institute of Agronomic Research, FRANCE

## Abstract

**Background:**

The burden of obesity in Vietnam has not been well defined because there is a lack of reference data for percent body fat (PBF) in Asians. This study sought to define the relationship between PBF and body mass index (BMI) in the Vietnamese population.

**Methods:**

The study was designed as a comparative cross-sectional investigation that involved 1217 individuals of Vietnamese background (862 women) aged 20 years and older (average age 47 yr) who were randomly selected from the general population in Ho Chi Minh City. Lean mass (LM) and fat mass (FM) were measured by DXA (Hologic QDR 4500). PBF was derived as FM over body weight.

**Results:**

Based on BMI ≥30, the prevalence of obesity was 1.1% and 1.3% for men and women, respectively. The prevalence of overweight and obesity combined (BMI ≥25) was ~24% and ~19% in men and women, respectively. Based on the quadratic relationship between BMI and PBF, the approximate PBF corresponding to the BMI threshold of 30 (obese) was 30.5 in men and 41 in women. Using the criteria of PBF >30 in men and PBF >40 in women, approximately 15% of men and women were considered obese.

**Conclusion:**

These data suggest that body mass index underestimates the prevalence of obesity. We suggest that a PBF >30 in men or PBF >40 in women is used as criteria for the diagnosis of obesity in Vietnamese adults. Using these criteria, 15% of Vietnamese adults in Ho Chi Minh City was considered obese.

## Introduction

Obesity is recognized as a global health problem because it affects a large proportion of individuals in developed and developing countries [1, 2]. In the United States, 32% of adult men and 35% of adult women are obese (ie BMI ≥ 30 kg/m^2^) [3]. In Asia, approximately 17% of population is considered obese by World Health Organization (WHO) [4]. Obesity is associated with an increased risk of mortality [5], type 2 diabetes [6], cardiovascular diseases and cancer [7]. Moreover, obese individuals have 7 times higher the risk of **developing** diabetes than individuals of a normal BMI [8]. Since obesity is increased with advancing age, the on-going rapid aging of the population will further impose a greater burden on the society. Indeed, it has been estimated that by 2030 nearly one-third of the world population is overweight or obese [1].

Although it is believed that obesity is increasing in Asian populations, there is actually no consensus on the definition of obesity for Asians. In 2004, a WHO expert consultation concluded that Asian individuals are at greater risk of type 2 diabetes and cardiovascular disease with a lower BMI than their Caucasian counterparts, but the consultation did not come up with a consensus cut-off BMI for defining obesity in Asians [9]. The consultation also proposed that the WHO BMI cut-off points should be retained as international classifications. In reality, some groups use the BMI ≥25 or BMI ≥27.5 as a cut-off value for the diagnosis of obesity in Asian men and women [10, 11].

Clinically, obesity is defined as the accumulation of excess body fat to the extent that it may have adverse effects on health. It is crucial to determine a threshold of body fat that is associated with potential harm to an individual’s health. In the absence of body fat measurement, the ratio of weight over height squared or body mass index (BMI), also referred to as Quetelet index, is a common and useful indicator for defining obesity in adult individuals [12]. However, it is increasingly recognized that fat mass, rather than BMI, is a better indicator of true fat mass and hence obesity. Weight is primarily made up of fat mass and muscle mass. BMI, with weight in the numerator, can not distinguish between the two components. Thus, an individual with high muscle mass can be classified as obese, even though the individual does not carry excess body fat. By the clinical definition, a better measure of obesity should be based on an individual’s percent body fat (PBF), which can now be measured by a variety of instruments, including bioelectrical impedance analysis, magnetic resonance imaging, computed tomography, and dual energy X-ray absorptiometry (DXA).

While the WHO recommended BMI thresholds for defining obesity and overweight are well established, it is not clear what is the appropriate threshold of PBF for classifying an individual as obese. It is widely claimed that a PBF greater than 25% for men and 35% for women are the criteria for diagnosing obesity [13–18]. The claim is attributed to a WHO report, but we have pointed out that this claim is a misquotation of the WHO Technical Report [19], which makes no recommendation of any PBF threshold. As a matter of fact, until now there exist no body fat thresholds for defining obesity.

It has been assumed that for a given BMI, Asians have greater PBF than Caucasians [20, 21]. However, a close examination of the data on which this assumption is based on[21] reveals little difference in PBF between Chinese in New York and Caucasian women. We have previously shown that after matching for BMI, Vietnamese women and American white women have virtually identical PBF [22]. Thus, it appears that there is no sound justification for lowering BMI criteria for defining obesity in Asians.

Vietnam is a developing country with a population of approximately 92 million [23], representing 1.3% of the world population. Approximately 70% of the population lives in rural areas. During the past 20 years, the country has continued to be one of the world's fastest economic growth, with annual growth rate of ~5% [24]. Parallel with the economic development, Vietnam has also undergone remarkable changes in dietary patterns [25] which led to a change in BMI [26]. Therefore, the population is an ideal setting for studying the burden of obesity in transitional economies. However, no studies in the past have examined the burden of obesity using PBF as an indicator in Vietnam. Thus, in this study, we sought to analyze the association between PBF and BMI, and to define the prevalence of overweight and obesity using both BMI and PBF criteria.

## Study Design and Methods

### Study setting and population

The study was conducted in Ho Chi Minh City, the largest city in Vietnam. The city is also a major economic hub, with a population of 7.8 million (Vietnam General Statistics Office, 27/3/2015). The recruitment of participants and data collection had taken place between February 2010 and December 2010. The study was conducted in accordance with the principles of medical ethics of the World Health Organization. All participants were provided with full information about the study's purposes, and gave written informed consent to participate in the study. The research protocol and procedures were approved by the Scientific Committee of the People's Hospital 115 and Pham Ngoc Thach University of Medicine.

Details of study procedures have been published elsewhere [27, 28]. Briefly, the study was designed as a cross-sectional investigation, in which individuals were sampled from the general population according to a random sampling scheme. We approached community organizations, including churches and temples, in each district to obtain the list of members aged 18 years and above, and this list was served as a sampling frame for the study. Next, we use an R program package to randomly select individuals aged 18 years or above, and the selected individuals were contacted to invite to participate in the study. About 5% of the invited individuals did not respond to our letter, and they were invited via phone. The participants did not receive any financial incentive, but they received a free health check-up, including lipid and blood glucose analyses. Participants were excluded from the study if they had rheumatoid arthritis.

### Measurements and data collection

Data collection was done by direct interview and direct measurement. Upon signed the informed consent form, participants were administered a structured questionnaire that collected data concerning anthropometry, lifestyle, and clinical history. Each participant was asked to provide information on current and past smoking habits. Smoking status and alcohol use (current, past, and never) was ascertained by the questionnaire. Clinical data including blood pressure, pulse, and reproductive history (i.e. parity, age of menarche, and age of menopause), medical history (i.e. previous fracture, previous and current use of pharmacological therapies) were also obtained. Body weight was measured on an electronic scale with indoor clothing without shoes. Height was determined without shoes on a portable stadiometer with mandible plane parallel to the floor. Body mass index (BMI) was calculated as weight in kg over height in meter squared.

All participants underwent a DXA scan of the whole body (Hologic QDR 4500, Hologic, Inc., Bedford, MA, USA). Body composition, including lean mass, fat mass and bone mineral content, was obtained from the scan. The densitometer was standardized by a standard phantom before each measurement was undertaken. Fat mass was expressed in kilogram as well as in percentage of body weight.

In addition, in order to adjust for body height, we fitted the equation of log FM or log LM against height: log(FM) = *k* + *a*×log(height), and log(LM) = *c* + *b*×log(height). Using the observed data from our study, we found that *a =* 1.96 and *b* = 1.70, which is close to 2. Therefore, we derived the fat mass index (FMi) and lean mass index (LMi) by the following formulae: FMi = FM / (height)^2^ and LMi = LM / (height)^2^, which is interestingly similar to the calculation of body mass index [29].

### Data analysis

The relationship between PBF and BMI was analyzed by a Bayesian multiple linear regression model. In the model, PBF was considered the dependent variable; BMI, age, and gender were independent variables. In exploratory analysis, we found that the relationship between PBF and BMI was not linear, and a quadratic model was appropriate. Thus, the model was PBF = α + β_1_×Gender + β_2_×Age + β_3_×BMI + β_4_×BMI^2^, in which α and β coefficients were estimated by observed data. The uniform prior was used in the regression model by placing equal likelihood to all possible values of the regression coefficient can take. The assumptions of the linear regression (i.e. normal distribution, homogeneity and independent errors) were satisfied by residual analysis. All analyses were conducted with the R statistical language [30] and the Bayesian analyses were performed with the MCMC package [31].

## Results

The study included 355 men and 862 women aged 20 years and above. The average age was 44 (SD 19) and 49 (SD16) for men and women, respectively. As expected, men had lower fat mass and PBF, but greater lean body mass and bone density than women. The difference in PBF between men and women was almost 2 SDs. Almost 45% of men and 1% of women self-reported as past and current smokers (**Table 1**).

**Table 1:** Anthropometric characteristics and lifestyle factors of study participants.

Variable	Men	Women	P-value
Number of participants	355	862	
Age (yr)	43.7 (18.8)	48.6 (16.4)	<0.0001
Number of participants by age group			
18–29	107	134	
30–39	46	95	
40–49	54	209	
50–59	74	212	
60+	74	212	
Weight (kg)	62.0 (9.5)	52.3 (7.7)	<0.0001
Height (cm)	165.1 (6.7)	153.3 (5.6)	<0.0001
Body mass index (kg/m^2^)	22.7 (3.0)	22.3 (3.1)	0.013
Whole body fat mass (kg)	15.0 (5.1)	18.2 (5.0)	<0.0001
Percent body fat (%)	24.2 (5.8)	34.7 (5.2)	<0.0001
Whole body lean mass (kg)	43.8 (5.8)	32.0 (4.0)	<0.0001
Whole body bone density (g/cm^2^)	1.06 (0.10)	0.98 (0.11)	<0.0001
Current smokers (n; %)	159 (44.8)	7 (0.8)	<0.0001
Alcohol users (n; %)	182 (51.3)	29 (3.4)	<0.0001

Note: Values are mean and standard deviation (in brackets), or actual number of participants and gender-specific percentage (in brackets).

Descriptive analyses of fat mass and lean mass by 10-year age group are shown in **Table 2**. PBF increased with advancing age, and the rate of increase was greater in women than in men. By the age of 60 or above, PBF in men was 26% (SD 5.9%) and in women 37% (SD 5.3%). Even after correcting for height, fat mass index still showed a downward trend with advancing age. There was a divergent trend in lean mass between men and women. In men, there was an age-associated decline in lean mass, with the rate of decline being 0.11 kg/year. However, in women, there was no significant change in lean mass with age.

**Table 2:** Mean and standard deviation of percent fat mass, fat mass, and lean mass in 355 men and 862 women classified by age group.

Age group	Men (n = 355)	Women (n = 862)
**Percent body fat**		
18–29	23.17 (6.21)	31.18 (4.70)
30–39	22.17 (5.54)	32.91 (4.82)
40–49	24.34 (5.12)	34.46 (4.63)
50–59	24.85 (5.01)	35.61 (4.62)
60+	25.99 (5.86)	36.94 (5.27)
**Fat mass (kg)**		
18–29	15.05 (5.95)	15.20 (4.12)
30–39	13.36 (4.80)	16.63 (4.61)
40–49	15.43 (4.43)	18.42 (4.42)
50–59	15.58 (4.62)	19.41 (4.82)
60+	14.94 (4.83)	19.53 (5.24)
**Fat mass index (kg/m** ^**2**^ **)**		
18–29	5.15 (1.94)	6.23 (1.56)
30–39	5.02 (1.74)	7.02 (1.89)
40–49	5.78 (1.67)	7.79 (1.85)
50–59	5.79 (1.68)	8.19 (2.01)
60+	5.77 (1.80)	8.64 (2.21)
**Lean mass (kg)**		
18–29	46.00 (5.61)	31.30 (3.99)
30–39	43.48 (5.28)	31.45 (4.04)
40–49	45.21 (6.11)	32.75 (3.68)
50–59	43.92 (5.06)	32.85 (3.96)
60+	39.49 (4.59)	31.28 (3.96)
**Lean mass index (kg/m** ^**2**^ **)**		
18–29	15.79 (1.56)	12.84 (1.26)
30–39	16.37 (1.44)	13.28 (1.52)
40–49	16.89 (2.19)	13.84 (1.33)
50–59	16.35 (1.69)	13.86 (1.44)
60+	15.27 (1.55)	13.85 (1.47)
**Body mass index (kg/m** ^**2**^ **)**		
18–29	22.08 (2.89)	20.00 (2.18)
30–39	22.66 (2.66)	21.11 (2.54)
40–49	23.92 (3.37)	22.54 (2.75)
50–59	23.34 (2.98)	23.04 (3.08)
60+	22.25 (3.00)	23.13 (3.15)

Note: Values shown are actual number of individuals and gender-specific percentages. Fat mass index and lean mass index were calculated as actual mass (in kg) per height in meter squared.

### Prevalence of overweight and obesity based on BMI

Approximately 9% of adult men and women were classified as underweight (i.e., BMI less than 18.5, **Table 3**). Using BMI ≥25.0 criteria, approximately 24% of men and 19% of women were classified as overweight or obese. However, only 1.1% of men and 1.3% of women were obese. If the BMI ≥27.5 criteria were used, 6.5% of men and 4.9% of women were considered obese.

**Table 3:** Prevalence of underweight, overweight and obesity by various BMI criteria.

BMI Criteria	Men (n = 355)	Women (n = 862)
<18.5	31 (8.7)	76 (8.8)
18.5 to 24.9	239 (67.3)	626 (72.6)
25.0 to 29.9	81 (22.8)	149 (17.3)
30 and above	4 (1.1)	11 (1.3)

Note: Values shown are actual number of individuals and gender-specific percentages.

The prevalence of overweight and obesity was increased with age. Among those aged less than 30 years, only 1.5% of women were classified as overweight or obese compared with ~15% of men. Among those aged 60 years and older, almost 30% of women and 24% of men were overweight or obese (data not shown).

### Relationship between PBF and BMI

**Fig 1:**
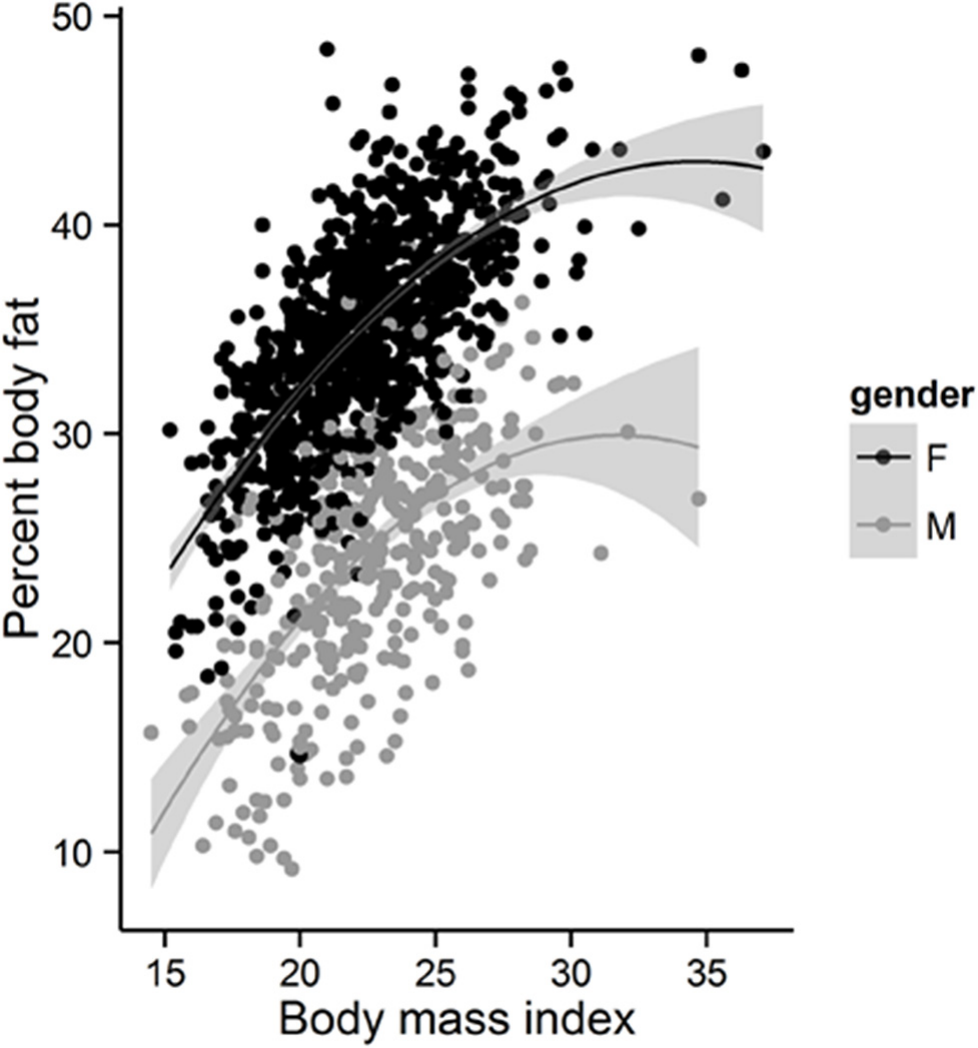
Relationship between BMI and PBF (%) for men (grey dots) and women (black dots).

In either gender, the relationship between BMI and PBF could be described by a quadratic regression model. Accordingly, among individuals with BMI <27, PBF was linearly related to BMI; but among those with BMI >27 the relationship between the two variables were leveled off (**Fig 1**). In addition, age and gender were also statistically associated with PBF (**Table 4**). After adjusting for age and BMI, men on average had PBF lower than women by ~11% (95% CrI, 10.4 to 11.4%). In either men or women, each year increase in age was associated with 0.04% (95% CrI, 0.03 to 0.06%) increase in PBF. Collectively, the 3 factors (ie age, gender, and BMI) explained ~70% of the variation in PBF among individuals. An alternative model for describing PBF is shown in **Table 4**, where age is replaced by lean mass. According to this model (with R-squared value being 0.77), each kg increase in lean mass was associated with a 0.55% decrease in PBF, and this effect was independent of gender.

**Table 4:** Bayesian regression models for predicting percent body fat.

Predictor	Regression coefficients	95% Credible internal
**Model I: Predictors are gender, age, and BMI**		
Intercept	-18.9	-26.7 to -11.2
Sex (men)	-10.9	-11.4 to -10.4
Age (yr)	0.044	0.031 to 0.057
Body mass index (BMI)	3.473	2.812 to 4.153
BMI squared	-0.051	-0.066 to -0.037
**Model II: Predictors are gender, lean mass, and BMI**		
Intercept	-13.1	-20.0 to -6.2
Sex (men)	-9.0	-12.2 to -5.8
Lean mass (kg)	-0.55	-0.6 to -0.5
Body mass index (BMI)	4.20	3.6 to 4.8
BMI squared	-0.056	-0.069 to -0.043
BMI x Sex(men)	0.18	0.03 to 0.32

Notes: For model I, the coefficient of determination (R^2^) was 0.71, with variance being 15.2 (95% CI, 14.0 to 16.5). For model II, the R^2^ was 0.77, and variance was 12.0 (95% CI, 11.0 to 13.0).

**Fig 2:**
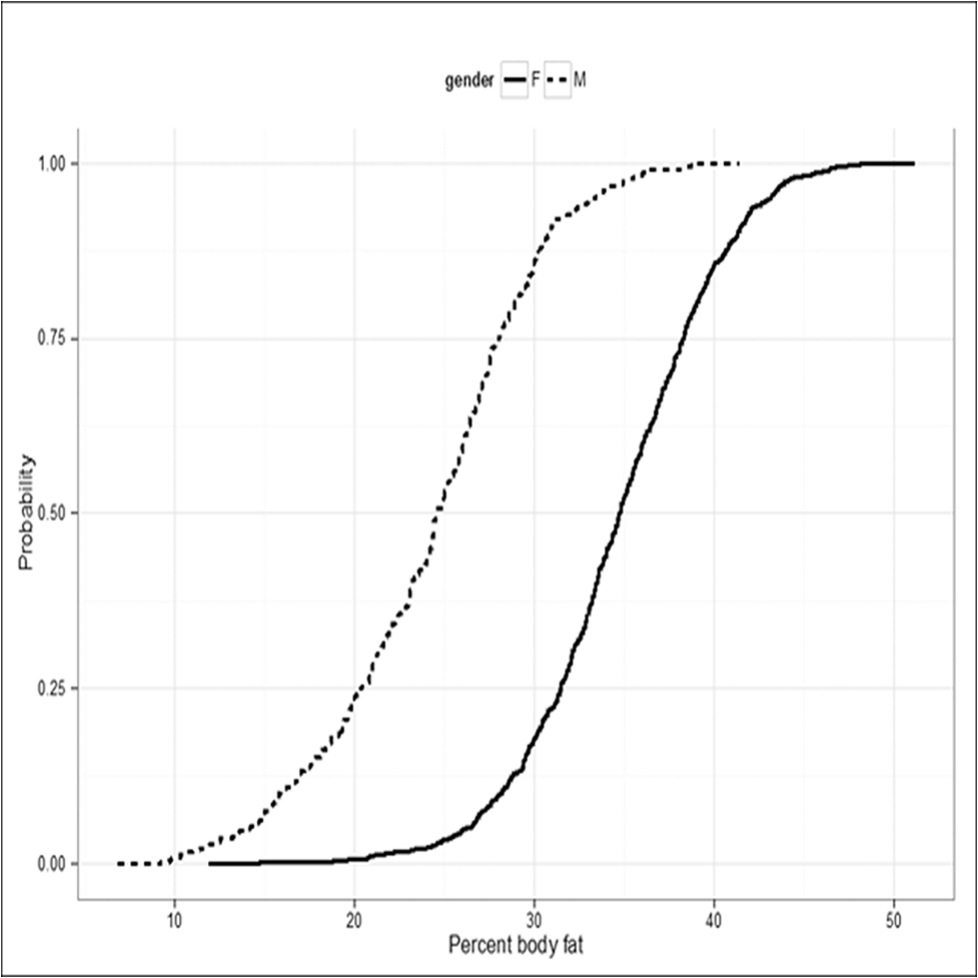
Cumulative distribution of percent body fat for men (dotted line) and women (solid line).

Based on the estimated parameters of model I, we derived the PBF values corresponding to a BMI for men and women aged 50 years (**Table 5**). In men, the approximate PBF threshold corresponding to BMI of 18.5, 25, and 30 kg/m^2^ were 19.1, 27.2, and 30.5, respectively. The corresponding approximate PBF values for women were 30.0, 38.1, and 41.4, respectively. **Fig 2**shows the cumulative distribution of PBF for men and women. It can be estimated from the figure that approximately 14% of men had PBF greater than 30, and ~15% of women had PBF greater than 40.

**Table 5:** Mean and 95% confidence interval of percent body fat corresponding to a BMI threshold for men and women aged 40, 50, and 60 years.

Gender and BMI	Predicted percent body fat by age		
	40 yr	50 yr	60 yr
**Men**			
18.5 kg/m^2^	**18.7** (11.0, 26.4)	**19.1** (11.5, 26.8)	**19.6** (11.9, 27.3)
25.0 kg/m^2^	**26.9** (19.1, 34.5)	**27.2** (19.6, 34.9)	**27.7** (20.0, 35.3)
30.0 kg/m^2^	**30.1** (22.4, 37.8)	**30.5** (22.8, 38.2)	**30.9** (23.2, 38.6)
**Women**			
18.5 kg/m^2^	**29.6** (21.9, 37.2)	**30.0** (22.4, 37.7)	**30.5** (22.8, 38.1)
25.0 kg/m^2^	**37.7** (30.0, 45.3)	**38.1** (30.4, 45.8)	**38.5** (30.9, 46.2)
30.0 kg/m^2^	**40.9** (33.2, 48.6)	**41.4** (33.7, 49.1)	**41.8** (34.1, 49.5)

Note: Values were derived from model I: PBF = -18.9–10.9×(sex = men) + 0.044×Age + 3.473×BMI − 0.051×BMI×BMI.

## Discussion

Vietnam is a country in the middle of transition from an agrarian society to industrialized society with middle level income. The country is, thus, an ideal setting to study the burden of overweight and obesity which has not been well documented. In this study, we have shown that using BMI, the prevalence of obesity was still low (around 1%), but the prevalence of overweight and obesity was 20%. However, using PBF>30 (in men) and PBF>40 (in women), the prevalence of obesity was ~15%.

The diagnosis of obesity has been largely based on BMI. Based on the relationship between BMI and mortality risk in Caucasian population, it was proposed that an adult who had BMI of 30 or greater is considered obese [32]. However, some argued that such a threshold may not be applicable to Asians, because there were observations that Asian people tended to have type 2 diabetes and cardiovascular disease at BMI levels lower than that in Caucasian people [16, 33]. Some experts proposed that the criteria for defining obesity in Asian populations should be lowered to BMI 27.5 kg/m^2^ [32]. Nevertheless, recent studies on the association between BMI and mortality in Asians have shown that people with BMI in the range of 18.5 to 25.0 have lowest mortality risk, and the risk was elevated when BMI exceeds 30 [34, 35]. Studies in Taiwan [36], China [35] and Singapore [37] all showed that the relationship between BMI and mortality followed a similar pattern of those observed in Caucasian populations. Thus, it appears reasonable that a BMI ≥25 and ≥30 kg/m^2^ can be considered "overweight" and "obese" (or at higher risk of death), respectively.

By using this criteria (BMI ≥25 kg/m^2^), we found that one-fifth of the Vietnamese adult population is overweight or obese. This prevalence is slightly lower than those observed in China [38], Thailand [39], Malaysia [40]. In China, the prevalence of overweight and obesity was 27% in men and 31% in women [38]. In Thailand, the country with lower per-capita income than China, approximately a-quarter of the adult population was overweight or obese, with the prevalence of obesity being ~5% [39]. In Malaysia, the country with better per-capita income than China and Thailand, the prevalence estimates for overweight (34%) and obesity (19.5%) are approaching the prevalence observed in Western populations [40]. Collectively, these data appear to suggest that the prevalence of obesity varies proportionately with incomes or levels of economic development. As Vietnam is approaching the status of a middle income country, it can be predicted that the burden of overweight and obesity is increasing in the near future. Indeed, national survey data showed that the prevalence of overweight and obesity (BMI ≥25) in 2005 (7%) was almost double the rate in 2000 (3.7%).

Although overweight/obesity is a serious concern, underweight is another public health problem in Vietnam. In our study, we found that almost 9% of adult men and women are underweight. This prevalence is only half of a previous national estimate which was 20.9% [41]. However, our study sampled adult people from Ho Chi Minh City (formerly Saigon) which is a urban population, and it is expected that the prevalence of underweight in rural areas is even higher than that in urban areas.

Although BMI is a practical measure, it is clearly not an ideal indicator of obesity. The main reason is that BMI, being a crude measure, could not distinguish between fat mass and lean mass. A body composition assessment which could accurately measure the amount of whole body fat mass (and hence PBF) is an ideal indicator of obesity. For approximately 20 years, it has been assumed that for a given BMI level, Asian people have greater fat mass than Caucasian people [14, 16, 21, 42, 43]. However, in a study comparing PBF between Vietnamese and White American women, we found no difference in PBF between the two groups even after matching for BMI [22]. In this study, we also note that the gender-and-age-specific PBF was lower than that observed in the White population [44]. Thus, again there is no good rationale for lowering BMI threshold for defining obesity in Asians.

While the WHO recommended BMI thresholds for defining obesity and overweight are well established, it is not clear what is an appropriate threshold of PBF for classifying an individual as obese. Many investigators have previously asserted that obesity is defined as a PBF greater than 25% for men and 35% for women, because the thresholds are believed to correspond to a BMI of 30 kg/m^2^ in young Caucasians[45], and attributed this fact to a WHO Technical Report [19]. As a result, the PBF thresholds have been used widely as a rationale for conducting studies into the validity of BMI. However, in reality, the WHO Technical Report makes no recommendation of any PBF threshold for defining obesity, and we have raised this point [46]. Moreover, the WHO Report [19] nowhere states that a PBF of 25% in men and 35% in women corresponds to a BMI of 30 kg/m^2^. The attribution to WHO Technical Report is, thus, a misquotation, and many research in this particular area (ie PBF and obesity) over the past 10 years has been built on an incorrect referencing.

The best and most appropriate approach to define a threshold for obesity is based on the relationship between PBF and “hard” outcome such as mortality in long-term prospective study which has not been done. Thus, an alternative approach is required. In a previous study based on the Korean population, investigators used CVD as an outcome, and they found that the “optimal” PBF cutoffs were 21% for men and 37% for women [47]. With these thresholds, the investigators estimated that the prevalence of obesity in men and women was 42% and 16%, respectively [47], a large discrepancy. If we were based on these criteria, the prevalence of obesity in our population would be 73% for men and 34% for women, which appear to be unreasonable.

In this study, based on the relationship between PBF and BMI, we have derived PBF thresholds corresponding to underweight (BMI 18.5), overweight (BMI 25) and obese (BMI 30). We found that a PBF of ~30 in men and 40 in women aged 50 years are corresponding to the BMI of 30. Using the criteria of PBF >30 in men and PBF >40 in women, approximately 14% of men and 15% of women were considered overweight. These estimates are higher than those based on BMI ≥30 criteria, but this difference is expected because for a given BMI level, there is a considerable variation in PBF. In other words, BMI considerably underestimated the prevalence of obesity in this population, and this finding is consistent with previous studies in Caucasian populations [48, 49].

The present study’s findings must be interpreted within context of strengths and weaknesses. The study was based on a reasonably large sample size, and participants were randomly selected from the general population to ensure its representativeness and external validity. The use of DXA technology is a strength, because the technology is considered “gold standard” for measuring body composition parameters. The study is the first to provide reference data for DXA-based body composition in the Vietnamese population. However, a number of potential weaknesses should also be noted. The study was designed as a cross-sectional investigation, and it is therefore not possible to make any causal inference on the relationship between PBF and BMI. Our participants were sampled from a urban setting; therefore, our results may not be generalizable to the general population in Vietnam where there is ~70% of population living in rural areas. Nevertheless, our results provide an intriguing glimpse into the double burden of underweight and overweight in a population that is undergoing rapid transition, and is thus a good reference for future studies in transitional populations.

In conclusion, these data suggest that body mass index underestimates the prevalence of obesity in the Vietnamese population. We propose that a percent body fat >30 in men or PBF >40 in women is used as criteria for the diagnosis of obesity in Vietnamese adults. Using these criteria, 15% of Vietnamese adults in Ho Chi Minh City was considered obese.
